# Characterization of an Etch Profile at a Wafer Edge in Capacitively Coupled Plasma

**DOI:** 10.3390/nano12223963

**Published:** 2022-11-10

**Authors:** Inho Seong, Jinho Lee, Sijun Kim, Youngseok Lee, Chulhee Cho, Jangjae Lee, Wonnyoung Jeong, Yebin You, Shinjae You

**Affiliations:** 1 Applied Physics Lab for PLasma Engineering (APPLE), Department of Physics, Chungnam National University, Daejeon 34134, Korea; 2 PSK, Hwaseong-si 18449, Korea; 3 Samsung Electronics, Hwaseong-si 18448, Korea; 4 Institute of Quantum Systems (IQS), Chungnam National University, Daejeon 34134, Korea

**Keywords:** plasma, sheath, etching distortion, database

## Abstract

Recently, the uniformity in the wafer edge area that is normally abandoned in the fabrication process has become important for improving the process yield. The wafer edge structure normally has a difference of height between wafer and electrode, which can result in a sheath bend, distorting important parameters of the etch, such as ionic properties, resulting in nonuniform etching. This problem nowadays is resolved by introducing the supplemented structure called a focus ring on the periphery of the wafer. However, the focus ring is known to be easily eroded by the bombardment of high-energy ions, resulting in etch nonuniformity again, so that the focus ring is a consumable part and must be replaced periodically. Because of this issue, there are many simulation studies being conducted on the correlation between the sheath structural characteristics and materials of focus rings to find the replacement period, but the experimental data and an analysis based on this are not sufficient yet. In this study, in order to experimentally investigate the etching characteristics of the wafer edge area according to the sheath structure of the wafer edge, the etching was performed by increasing the wafer height (thickness) in the wafer edge area. The result shows that the degree of tilt in the etch profile at the wafer edge and the area where the tilt is observed severely are increased with the height difference between the wafer and electrode. This study is expected to provide a database for the characteristics of the etching at the wafer edge and useful information regarding the tolerance of the height difference for untilted etch profile and the replacement period of the etch ring.

## 1. Introduction

In modern semiconductor manufacturing, dry processing that uses plasma, such as deposition and etching, has attracted enormous interest for the ability to shrink the feature size and the structure of semiconductors in response to the increased demand for improved computational capability [1]. Although wet processing using chemical liquid has also attracted interest due to its relatively fast process and simple equipment, it is known as isotropic etching because the corrosive action occurs in all directions [2,3]. Therefore, in particular, low-temperature plasma technology is required for anisotropic high-level etch processing such as deep-and-narrow contact hole etching [4,5,6,7,8,9,10]. Because this process is sensitive to even a small change in plasma parameters, research on the chamber structure or process recipe has been continuously conducted to overcome this problem [9,10,11,12,13,14,15,16]. Nevertheless, in high aspect ratio contact hole etching, which requires high-ion energy, there are still many defects that damage chamber components. These parts are constantly damaged, causing defects that have a major impact on yield. Therefore, their damaged parts are periodically replaced as consumables.

Among the consumables, a focus ring, which is installed around the edge of the wafer, is necessary to improve the etch uniformity over the wafer area by extending the plasma above the wafer edge [17,18,19,20,21]. The focus ring is continuously exposed to high-energy ions and etched and eroded [22]. Therefore, due to the eroded focus ring, the plasma and sheath characteristic is distorted at the wafer edge, which deteriorates the processing yield. Therefore, research has been conducted on the correlation of the sheath and plasma between the focus ring and the wafer structure [21,23,24,25,26,27,28]. Babaeva et al. simultaneously investigated ion energy and angle distributions for the gap and height of the focus ring [21,23]. Kim et al. studied the ion kinetics at the wafer edge by varying the geometry of the focus ring in capacitively coupled plasma through particle simulation [25]. Kim et al. conducted the plasma molding over surface topography through simulation and measurement of ion energy and angle distributions over trenches [26,27]. Recently, Xiao et al. developed a one-dimensional circuit model with a focus ring and compared simulation results to experimental results [28].

In addition to the research on the correlation between the plasma sheath and focus ring, a lot of research has been conducted on the materials of the focus ring to improve the lifetime of the focus ring with less sputter yield. Yang et al. investigated the effect of the material and structure variation of the focus ring for enhanced etch resistance [29]. They focused on the formation of a smaller plasma sheath voltage over the focus ring to reduce the reactive ion energy bombardment. Kushner et al. computationally investigated the consequences of the dielectric constant of the focus ring materials on the erosion of the focus ring [30].

However, these studies were limited to simulations and investigated the characteristics of ions reaching the wafer surface according to the structural change of the focus ring. Therefore, the experimental etching characteristic data due to the structural step appearing at the wafer edge in the actual process is lacking. In previous studies [21,23,24,25,26,27,28], we confirmed that the height difference between the wafer and the focus ring rather than the height of the focus ring is a key factor in creating the sheath curvature. Therefore, we focused this key factor and experimentally simulated the sheath curvature via the change of the height difference between the wafer and electrode without the focus ring. In this situation, the etching was performed with variations of the height difference and the size of the contact hole. The experimental result shows that the degree of tilt in the etch profile at the wafer edge and the area where the tilt is observed severely are increased with the height difference between the wafer and electrode. The extended experiments for the hole size effect on the profile tilting and the analysis based on field simulation are also addressed in this paper.

## 2. Materials and Methods

**Figure 1:**
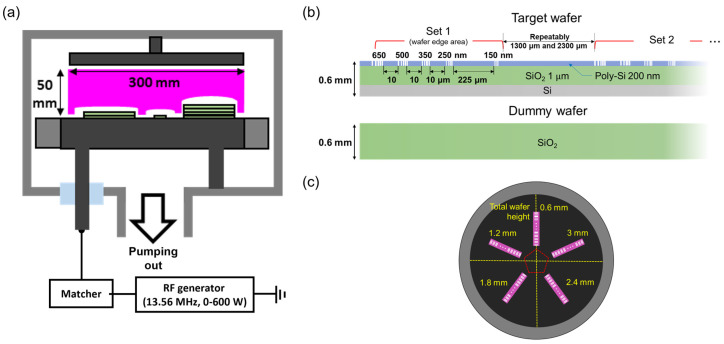
(**a**) A schematic of the experiment setup, (**b**) schematic showing the properties of a wafer, and (**c**) the top view of the powered electrode.

The experiments were conducted with Ar/C${}_{4}$F${}_{8}$ capacitively coupled plasma (CCP) source applied by 13.56 MHz RF power. Figure 1 shows a schematic of the CCP source that was employed for the etching process and a top view of the bottom electrode-placed wafers, and the features of the wafer. As can be seen in Figure 1a, this CCP chamber has a diameter of 300 mm and contains parallel plate electrodes separated by a distance of 50 mm. The top electrode, embedded in a showerhead to distribute the gas flow uniformly, is grounded, and the bottom electrode is connected to the 13.56 MHz RF power generator via an L-type matcher. The vacuum system composed of a rotary pump and turbo molecular pump maintains the base pressure at levels of the order of 10${}^{-6}$ Torr.

As shown in Figure 1b, the wafers used in the etching process were defined by a small size of approximately 15 mm by 80 mm and consisted of a layer of SiO${}_{2}$ (1 μm) deposited on a silicon substrate. The SiO${}_{2}$ layer was masked by a 200-nm thick polysilicon nanosized hole patterned layer. Because the etch processing of different size of the contact hole has been performed in the actual process, if the distortion characteristics are different, depending on the size of the contact hole due to the height difference, useful information such as the replacement period of the focus ring can also change. Therefore, the contact holes patterned in the mask (650, 500, 350, 250, and 150 nm in diameter) were used to figure out the effect of the hole size. The total height of the wafer is 0.6 mm including the mask, the target SiO${}_{2}$, and the silicon substrate. Simulation studies show that the height difference from the wafer due to erosion of the focus ring causes the sheath to bend, altering the ion trajectory at the wafer edge, which can tilt the etching [21,23,24,25,26,27,28]. Therefore, because the difference in height between the wafer and the focus ring is an important parameter, a bare SiO${}_{2}$ wafer with a height of 0.6 mm was placed under the wafer to be etched, and by increasing the number of wafers below, the height is increased to determine the etching effect according to the height difference between the wafer and the electrode without the focus ring. These contact holes are patterned in descending order from 650 nm, and a group of these holes is arranged in the order of 20 in the coupon wafer. One group of these holes is defined as set 1, and there are 20 of these sets alternating at intervals of 1.3 mm and 2.3 mm from the wafer edge. Figure 1c shows the position of each wafer by height when the height of the wafer to be etched is adjusted by using bare SiO${}_{2}$. The wafers are equally placed 30 mm from the center of the electrode and positioned to increase in height at 6-mm intervals counterclockwise from the 12 o’clock position.

**Figure 2:**
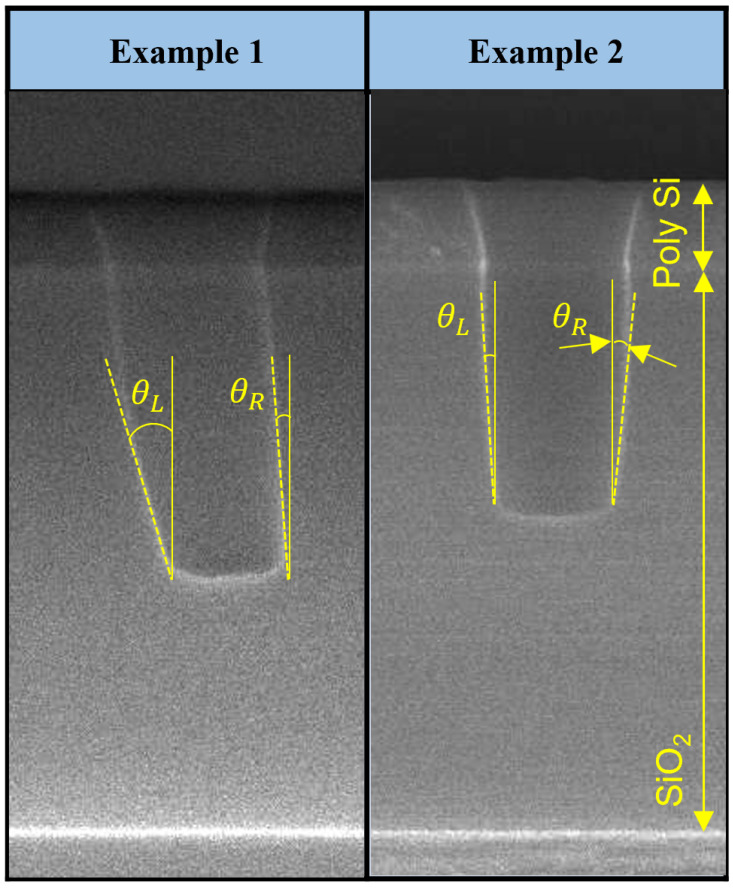
The definition of angles of the sidewall ($\theta_{L}$ and $\theta_{R}$).

The characteristics of etch profiles, such as the tilt of sidewall, were observed by cutting the etched coupon wafers into cross-sections by using scanning electron microscopy (SEM, TESCAN, Czechia). To quantitatively analyze these characteristics, each area of the etched contact hole was defined as sidewall and etch front. The right and left angles were defined as $\theta_{R}$ and $\theta_{L}$, which indicates the tilt relative to the normal vector of the etch front as depicted in Figure 2. With respect to the normal vector, counterclockwise and clockwise are defined as negative and positive, respectively. There are two examples in Figure 2; the first is the definition of each angle when the tapered portion is etched, and the second is when it is angled. The etching was performed for 10 min at the CCP power of 300 W, Ar flow rate of 65 sccm, C${}_{4}$F${}_{8}$ flow rate of 5 sccm, and pressure of 30 mTorr.

## 3. Results and Discussion

**Figure 3:**
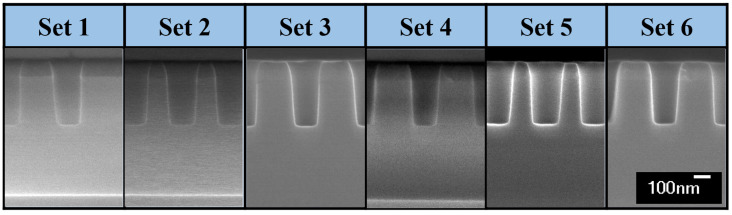
Cross-sectional SEM images of 250-nm contact holes each set point and in the 0.6 mm wafer.

Figure 3 shows cross-sectional SEM images of the etched contact holes as a function of the set number at the height of 0.6 mm. The set number is the closest one to the wafer edge, and the higher set number, the farther away from the edge. As shown in Figure 3, the etch profiles are presented up to the set 6 because at a higher set number from set 7, the result was not significantly different from the etch profiles in set 6. As can be seen in Figure 3, except for the case of set 1, the etch profile at the other set number is not tilted. In the process of using the CF-based gases, active species with isotropic motion in the hole typically form a passivating film in the sidewall [31,32,33,34,35]. As a result of the passivating film to prevent sidewall etching, the tapered etch profiles were obtained in which the magnitudes of $\theta_{R}$ and $\theta_{L}$ are equal but opposite in sign. On the other hand, in the etch profile in set 1, $\theta_{R}$ is smaller than $\theta_{R}$ in sets 5 and 6 far from the wafer edge, and $\theta_{L}$ is larger as a result of the difference in height of the wafer edges.

**Figure 4:**
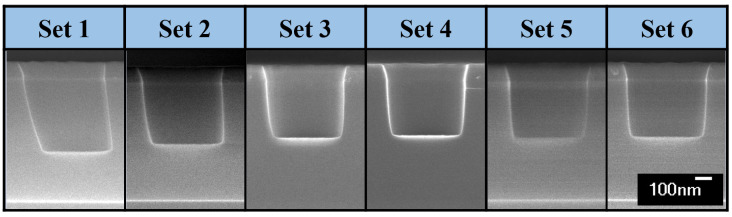
Cross-sectional SEM images of 650 nm contact holes each set point and in 3.0 mm wafer height.

Figure 4 shows cross-sectional SEM images of the etched contact holes as a function of the set number at the wafer height of 3.0 mm. As the height increases, it can be seen that the sidewall is tilted more dramatically than that at 0.6 mm wafer height. The tilted etch profile gradually changes to a tapered profile until set 3. The tilted etch profile shows that the sheath curvature is greater when the wafer height increases. That is, ions accelerated by a strongly distorted electric field near the wafer edge arrive at the wafer surface in highly tilted trajectories [21,23,25,26,27]. The etching amount gradually decreases from set 1 to set 3, as shown in Figure 4, because the etching yield of ions bombarding with a tilt angle is greater than that of ions bombarding vertically.

**Figure 5:**
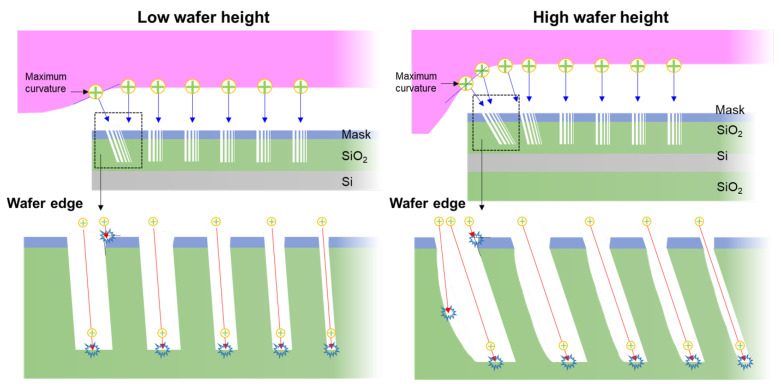
A schematic of the mechanism of the etch distortion according to the wafer height and contact hole size.

This is not the only etch characteristic of the ions appearing on the wafer edge. In set 1, shown in Figure 4, it can be seen that the profile of the left sidewall is curved and the right one is etched straight despite the fact that this profile does not appear in set 1 at the wafer height of 0.6 mm. Figure 5 shows a mechanism of the etch distortion according to the wafer height and contact hole size. At low height, ions in trajectories tilted at various angles are rare because of the small maximum curvature of the sheath at the wafer edge. Therefore, the profiles of the left and right sidewalls are the same. In contrast, at high height, there are ions in trajectories tilted at various angles due to the large maximum curvature of the sheath at the wafer edge. In the case of the left sidewall, the ions bombarding the wafer surface with various angles below the angle of the ion incident on the sheath boundary from the maximum curvature is also etched, whereas in the case of the right sidewall, the ions are blocked by the mask, resulting in etching at a different angle from the sidewall.

**Figure 6:**
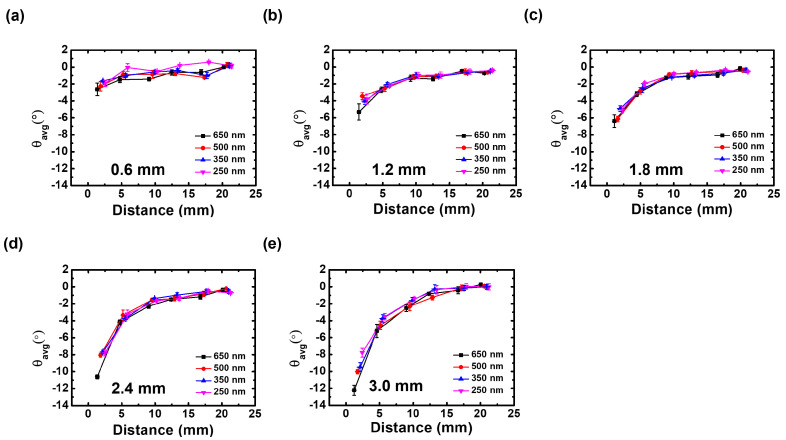
Averaged angles on left and right angle of the side wall with wafer heights. [(**a**) 0.6 mm, (**b**) 1.2 mm, (**c**) 1.8 mm, (**d**) 2.4 mm, (**e**) 3.0 mm].

**Figure 7:**
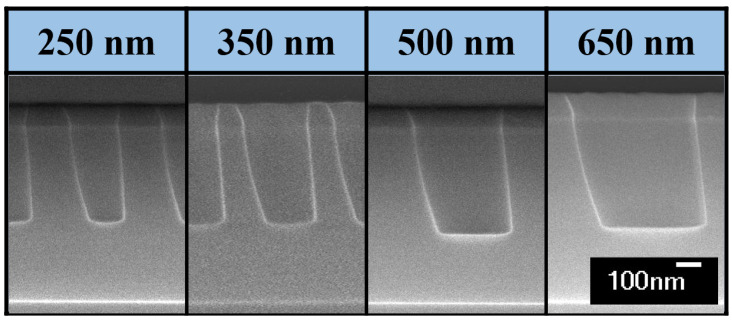
Cross-sectional SEM images of contact holes varying a diameter in set point 1 and 3.0 mm wafer height.

Figure 6 shows the difference between $\theta_{R}$ and $\theta_{L}$ depicted in Figure 2 for each wafer height and contact hole size to quantitatively analyze the degree of tilt of the etch profile up to set 6 (about 20 mm) based on the wafer edge at 0 mm. Figure 6a,e are the differences corresponding to the wafer height of 0.6 mm and 3.0 mm as shown in Figure 3 and Figure 4. As the height of the wafer increases, the tilt of the etch profiles appear farther. What is interesting here is that the tilt of the etch profile is highly dependent on the wafer height but is independent of the diameter of the contact hole. As shown in Figure 6 and Figure 7, the etching characteristics according to the contact hole size were investigated, but the 150-nm contact hole was omitted due to the limitation of the detection. The tendency independent of the contact hole appeared the same in any wafer height condition according to the change in the diameter of the contact hole. Because the ions are accelerated by the strong electric field in the sheath and incident on the wafer surface, the trajectory of the ions is similar to the direction of the electric field. If the size of the contact hole is patterned at a similar wafer position, ions enter the contact hole through the same tilted trajectory and are incident on the etch front as can be seen in Figure 5. Thus, because the characteristics of etching are determined by the characteristics of the ions, the size of the contact hole is irrelevant.

The deviation of the etch profile such as sidewall bowing can be caused by the lateral etching by bombardments of deviated ions [36]. The ion trajectories may deviate from ion reflection on the tilted mask. However, as shown in Figure 3 and Figure 4, the sidewall bowing at sets close to the wafer center was not observed despite that the mask wall is tilted. However, ions reflected from the mask at the wafer edge can create deviations in the etch profile. If the tilted incident ions collide with the mask tilted in the opposite direction, they may enter the contact hole and bombard the opposite sidewall. The left sidewall is curved as seen in the etch profile of set 1 in Figure 4. As mentioned in this paper, this etching can be performed by the effect of ions incident at the maximum curvature and smaller than that of the sheath. In addition, it may be due to ions reflected from the mask. Because this phenomenon is an etching characteristic that can sufficiently occur in an actual process, the results of this paper can represent the actual process.

**Figure 8:**
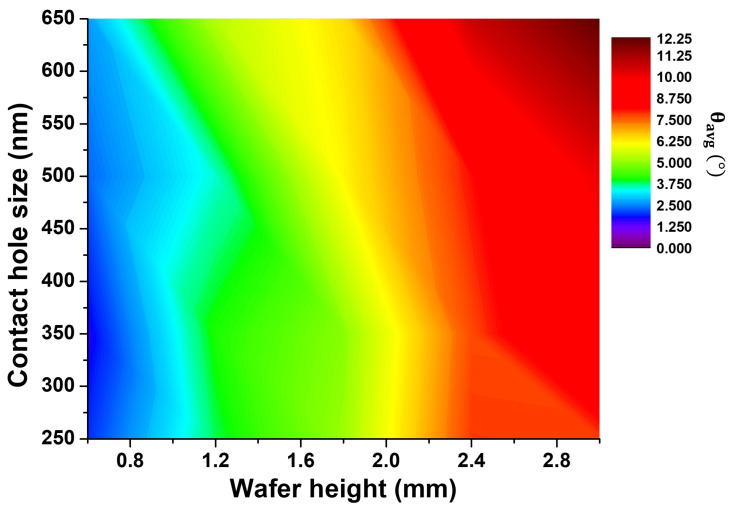
Contour map of the angle difference of the etched sidewall as functions of wafer height and contact hole size at the wafer edge.

The contour map in Figure 8 shows the angle difference between $\theta_{R}$ and $\theta_{L}$ as functions of wafer height and contact hole diameter at the wafer edge. As shown in Figure 7, at the wafer height of 0.6 mm or less, the tilt of the etch profile at the wafer edge is equal to or less than 2.5 degrees, and the tilt increases as the height of the wafer increases. Here, the wafer height means a step difference between the wafer and the electrode which might be a focus ring in real process. In a high aspect ratio etch process, a 2-degree tilt can be a defect, so the focus ring should be replaced before the 0.6-mm step is made. In addition, from this, when the wafer height is 0.6 mm or less, the yield at the wafer edge is increased without a focus ring, and economic loss due to consumables may be prevented.

## 4. Conclusions

In this study, we experimentally investigated the etch characteristics according to the curvature of the sheath due to the erosion of the focus ring. To simulate the curvature of the sheath, the etching wafer performed by increasing the height of the wafer. As a result, it was confirmed that the tilt of the etch profile at the wafer edge was strongly dependent on the wafer height. As the height of the wafer increased, the tilt of the etch profile at the wafer edge increased, and the region in which the tilt characteristics appeared also increased. As seen from the results of previous simulation studies, the etching characteristic is indicated by the curvature of the sheath due to the step difference between the wafer and the electrode and tilted trajectory of the ions caused therefrom. In addition, etching characteristics due to the direction of ions, which were not shown in previous studies, were observed. At the wafer edge, one sidewall is curved and the other one is etched straight. This result shows that not only ions with tilted trajectories but also ions reaching the wafer surface perpendicular to the wafer exist. Furthermore, as a result of investigating the etching characteristics according to the size of the contact hole, it was confirmed that it is independent of the size of the hole. This result shows that the ion trajectory determines the tilt of the etch profile.

This work presented the tilt map of the etching profile according to the wafer features at the wafer edge. At the wafer height of 0.6 mm or less, the tilt of the etch profile at the wafer edge is equal to or less than 2.5 degrees. In a high aspect ratio etch process, a 2-degree tilt can be a defect. Therefore, the focus ring should be replaced before the height difference of 0.6 mm between the wafer and the focus ring. In addition, if the wafer height is less than 0.6 mm, the tilt of the etch profile can be performed at less than 2.5 degrees in the etching process without a focus ring. This study will be able to provide a database on the etch distortion caused by the height difference between the wafer and the eroded focus ring at the wafer edge. In addition, it is expected to be a practical help for simulation analysis.

## Data Availability

The data presented in this study are available on request from the corresponding author.

## References

[1] Abe H., Yoneda M., Fujiwara N. (2008). Developments of plasma etching technology for fabricating semiconductor devices. Jpn. J. Appl. Phys..

[2] Shi D., Chen Y., Li Z., Dong S., Li L., Hou M., Liu H., Zhao S., Chen X., Wong C. (2022). Anisotropic Charge Transport Enabling High-Throughput and High-Aspect-Ratio Wet Etching of Silicon Carbide. Small Methods.

[3] Chen Y., Chen Y., Long J., Shi D., Chen X., Hou M., Gao J., Liu H., He Y., Fan B. (2021). Achieving a sub-10 nm nanopore array in silicon by metal-assisted chemical etching and machine learing. Int. J. Extrem. Manuf..

[4] Adamovich I. (2017). The 2017 Plasma Roadmap: Low temperature plasma science and technology. ESC J. Phys. D Appl. Phys..

[5] Oehrlein G.S., Metzler D., Li C. (2015). Atomic Etching at the Tipping Point: An Overview. ESC J. Solid State Sci. Technol..

[6] Kaler S.S., Lou Q., Donnelly V.M., Economou D.J. (2017). Atomic layer etching of silicon dioxide using alternating C_4_F_8_ and energetic Ar^+^ plasma beams. J. Phys. D Appl. Phys..

[7] Lee Y.S., Kim S.J., Lee J.J., Cho C.H., Seong I.H., You S.J. (2022). Purgeless atomic layer etching of SiO_2_. J. Phys. D Appl. Phys..

[8] Kasternmeier B.E.E., Matsuo P.H., Oehrlein G.S. (1999). Highly selective etching of silicon nitride over silicon and silicon dioxide. J. Vac. Sci. Technol. A.

[9] Suto S., Hayasaka N., Okano H. (1989). Highly Selective Etching of Si_3_N_4_ to SiO_2_ Employing Fluorine and Chlorine Atoms Generated by Microwave Discharge. J. Electrochem. Soc..

[10] Hayashi H., Kurihara K., Sekine M. (1996). Characterization of Highly Selective SiO_2_/Si_3_N_4_ Etching of high-Aspect-Ratio Holes. Jpn. J. Appl. Phys..

[11] Seman M., Wolden C.A. (2003). Investigation of the role of plasma conditions on the deposition rate and electrochromic performance of tungsten oxide thin films. J. Vac. Sci. Technol. A.

[12] Radjenovic B.M., Radmilovic-Radjenovic M.D., Petrovicm Z.L. (2008). Dynamics of the Profile Charging During SiO_2_ Etching in Plasma for High Aspect Ratio Trenches. IEEE Trans. Plasma Sci..

[13] Brichon P., Pujo E.D., Mourey O., Joubert O. (2015). Key plasma parameters for nanometric precision etching of Si films in chlorine discharge. J. Appl. Phys..

[14] Gopikishan S., Banerjee L., Bigkem K.A., Das A.K., Pathak A.P., Mahapatra S.K. (2016). Paschen curve approach to investigate electron density and deposition rate of Cu in magnetron sputtering system. Radiat. Eff. Defects Solids.

[15] Cho C.H., You K.H., Kim S.J., Lee Y.S., Lee J.J., You S.J. (2021). Characterization of SiO_2_ Etching Profiles in Pulse-Modulated Capacitively Coupled Plasmas. Materials.

[16] Im D.H., Min W.S., Hong S.J. (2020). Planar heating chuck to improve temperature uniformity of plasma processing equipment. Jpn. J. Appl. Phys..

[17] Yagisawa T., Shimada T., Makabe T. (2005). Modeling of radial uniformity at a wafer interface in a 2f-CCP for SiO_2_ etching. J. Vac. Sci. Technol. B.

[18] Uchida Y. (2015). Mounting Table Structure and Method of Holding Focus Ring. U.S. Patent.

[19] Panagopoulos T., Kim D., Midha V., Economou D.J. (2002). Three-dimensional simulation of an inductively coupled plasma reactor. J. Appl. Phys..

[20] Kubota M., Shima T. (2015). Focus Ring. U.S. Patent.

[21] Babaeva N.Y., Kushner M.J. (2007). Penetration of plasma into the wafer-focus ring gap in capacitively coupled plasmas. J. Appl. Phys..

[22] Koltonski M.E. (2015). Focus Ring Replacement Method for a Plasma Reactor, and Associated Systems and Methods. U.S. Patent.

[23] Babaeva N.Y., Kushner M.J. (2008). Ion energy and angular distributions into the wafer-focus ring gap in capacitively coupled discharges. J. Phys. D Appl. Phys..

[24] Tong L. (2015). Effects of gas composition, focus ring and blocking capacitor on capacitively coupled RF Ar/H_2_ plasmas. Jpn. J. Appl. Phys..

[25] Kim J.S., Hur M.Y., Kim H.J., Lee H.J. (2019). The ion kinetics at the wafer edge by the variation of geometry and permittivity of the focus ring in capacitively coupled discharges. J. Appl. Phys..

[26] Kim D., Economou D.J., Woodworth J.R., Miller P.A., Shul R.J., Aragon B.P., Hamilton T.W., Willison C.G. (2003). Plasma molding over surface topography: Simulation and measurement of ion fluxes, energies and angular distributions over trenches in RF high density plasmas. IEEE Trans. Plasma Sci..

[27] Kim D., Economou D.J. (2002). Plasma molding over surface topography: Simulation of ion flow, and energy and angular distributions over steps in RF high-density plasmas. IEEE Trans. Plasma. Sci..

[28] Xiao Y., Du Y., Smith C., Nam S.K., Lee H., Lee J.-Y., Shannon S. (2021). Focus ring geometry influence on wafer edge voltage distribution for plasma processes. J. Vac. Sci. Technol. A..

[29] Yang K.C., Park S.W., Lee H.S., Kim D.W., Yeom G.Y. (2017). Effect of structure and material variation of focus ring for enhanced etch resistance. Nanosci. Nanotechnol. Lett..

[30] Wang X., Lee H.-J., Nam S.K., Kushner M.J. (2021). Erosion of focus ring in capacitively coupled plasma etching reactor. J. Vac. Sci. Technol. A.

[31] Schwartz G.C., Rothman L.B., Schopen T.J. (1979). Competitive mechanisms in reactive ion etching in a CF_4_ plasma. J. Electrochem. Soc..

[32] Zhang D., Kushner M.J. (2001). Investigations of surface reactions during C_2_F_6_ plasma etching of SiO_2_ with equipment and feature scale models. J. Vac. Sci. Technol. A..

[33] Nagarajan R., Prasad K., Ebin L., Narayanan B. (2007). Development of dual-etch via tapering process for through-silicon interconnection. Sens. Actuators A.

[34] Huang S., Huard C., Shim S.-B., Nam S.-K., Song I.-C., Lu S., Kushner M.J. (2019). Plasma etching of high aspect ratio features in SiO_2_ using Ar/C_4_F_8_/O_2_ mixtures: A computational investigation. J. Vac. Sci. Technol. A.

[35] Sung D., Wen L., Tak H.-W., Lee H.-J., Kim D.-W., Yeom G.-Y. (2022). Investigation of SiO_2_ etch characteristics by C_6_F_6_/Ar/O_2_ plasmas generated using inductively coupled plasma and capacitively coupled plasma. Materials.

[36] Arnod J.C., Sawin H.H. (1991). Charging of pattern features during plasma etching. J. Appl. Phys..

